# Brief Research Report: Estimation of the Protein Digestibility-Corrected Amino Acid Score of Defatted Walnuts

**DOI:** 10.3389/fnut.2021.702857

**Published:** 2021-09-06

**Authors:** Kimberly A. Lackey, Stephen A. Fleming

**Affiliations:** Traverse Science, Inc., Champaign, IL, United States

**Keywords:** tree nuts, PDCAAS, protein, digestibility, walnuts

## Abstract

**Introduction:** Walnuts are considered a good source of essential fatty acids, which is unique among tree nuts. Walnuts are also composed of about 10–15% protein, but the quality of this protein has not been evaluated. Pistachios and almonds have been evaluated for their protein content using a protein digestibility-corrected amino acid score (PDCAAS), but it is unclear how the quality of protein in walnuts relates to that in other commonly consumed tree nuts. The objective of this study was to substantiate the protein quality of walnuts by determining their PDCAAS.

**Methods:** A small, 10-day dietary intervention trial was conducted using male Sprague-Dawley rats (*n* = 8, 4 per group) with two diets: a nitrogen-free diet and a diet containing protein exclusively from defatted walnuts. Feed intake and fecal output of nitrogen were measured to estimate the true protein digestibility, and the amino acid compositions of walnuts compared to child and adult populations were used to calculate amino acid scores (AAS) and PDCAAS.

**Results:** The true protein digestibility score of raw walnuts was calculated to be 86.22%. Raw walnuts contained 15.6 g protein/g walnut with AAS of 0.45 and 0.63 for children aged 6 months to 3 years and 3–10 years, respectively. For each population, a PDCAAS of 39 and 46% was calculated, respectively, using a protein conversion constant of 5.30. Using a protein constant of 6.25, a PDCAAS of 39% (6 months - 3 years) or 46% (3-10 years) was calculated.

**Conclusions:** This is the first known assessment of the PDCAAS of walnuts. Like almonds, they appear to have a low-to-moderate score, indicating they are not a quality source of protein.

## Introduction

Among tree nuts, walnuts represent a high-quality source of polyunsaturated fatty acids and are particularly high in α-linolenic and linoleic acid (1). Though walnuts may be unique in their lipid content, 10–15% of the composition of walnut is made up of protein (2), although the quality of this protein is unclear.

There are 20 amino acids that comprise human proteins; of these, nine are considered essential due to their required provision from dietary sources, rather than an endogenous source (3). Besides soy, high-quality protein sources tend to be found in animal products (4). However, it is widely recognized that plant sources of protein, including nuts and seeds, are also valuable sources of protein. With respect to tree nuts, lysine (Brazil nuts, cashews, hazelnuts, pine nuts, and walnuts), methionine and cysteine (almonds), and tryptophan (macadamias, pecans) are the limiting amino acids (5). To measure the quality of a protein source, a protein digestibility-corrected amino acid score (PDCAAS) can be calculated, which measures the quality of a protein-based on the amino acid requirements adjusted for digestibility as compared to a given reference population (e.g., children or adults).

The objective of this study was to determine the PDCAAS for walnuts such that the protein quality could be objectively evaluated against other tree nuts (namely, almonds and pistachios) and other sources of protein. To do this, a small, 10-day dietary intervention trial in rats was completed to estimate the true digestibility of nitrogen from walnuts for use in calculating the PDCAAS of walnuts. In this study, we estimated four PDCAAS using two conversion factors (5.30 and 6.25, discussed below) and for two reference populations {young children [Food and Agriculture Organization of the United Nations (FAO) scoring patterns for 6 months to 3 years of age] and older children and adults (FAO scoring pattern for 3–10 years of age)} such that the appropriate PDCAAS is available for multiple populations.

## Methods

### Animals

All animal care and study procedures complied with The Animal Welfare Act and were approved by the internal International Animal Care and Use Committee at Covance, Inc. (Princeton, NJ; protocol #8379737). The study was conducted at Covance test facilities in Greenfield, IN.

Eight male Sprague Dawley rats (21–28 days of age) were acquired from Envigo RMS, Inc. (“Envigo,” Indianapolis, IN) and allowed to acclimate to the test facility for 2 days prior to the start of the study. Rats were housed singly in polycarbonate caging with woodchip bedding and allowed *ad libitum* access to water and a standard rodent diet (Teklad Global Diet—Rodent 2014C; Envigo). Environmental conditions were controlled at a temperature of 20–26°C and relative humidity of 40–70% with 12-h light/dark cycles. Each animal was identified using a tail mark, microchip, and cage card. Animals were provided with cage enrichment devices. Animals were checked two times daily for mortality, clinical abnormalities, signs of pain, or distress. No clinical observations of adverse effects were noted throughout the trial; thus, all animals met the criteria for inclusion throughout the trial.

### Experimental Diets

Two experimental diets were formulated by Envigo. The nitrogen-free diet was an extremely low nitrogen modification of AIN-76A with no casein, additional starch to replace sucrose, and increased lipid (corn oil) content. The test diet (test) was formulated to contain 10% defatted walnuts as a protein source and to match the nitrogen-free diet with a 3.9 kcal metabolizable energy/g diet, 10% fat, 5% fiber, 3.5% AIN-76 mineral mix, 1% AIN-76A vitamin mix, and 0.2% choline bitartrate. Details of the composition of both diets can be found in Table 1. The diets were stored between 2 and 8°C until 2 h before administration each day. All diets and raw defatted walnut were analyzed for crude protein (AOCS Ac 4-91), fat (AOAC 948.22), total fiber (AOAC 985.29), and dry matter (DM) (AOAC 925.09).

**Table 1:** Composition of the experimental diets and defatted walnut.

**Formulated ingredients, g/kg**	**Nitrogen-free**	**Test**	
Defatted walnut	0	211.0	
Corn starch	653.0	484.41	
Maltodextrin	150.0	150.0	
Corn oil	100.0	94.15	
Cellulose	50.0	13.08	
Mineral mix (AIN-76 170915)	35.0	35.0	
Vitamin mix (AIN-76A 40077)	10.0	10.0	
Choline bitartrate	2.0	2.0	
**Analyzed nutrient composition, as-is basis**			
Metabolizable energy, g/kg	3.9	3.9	**Defatted walnut**
Crude protein (*N* ×5.30), g/100 g	0.29	10.41	47.45
Crude protein (*N* ×6.25), g/100 g	0.34	12.28	55.96
Nitrogen, g/100 g	0.05	1.97	8.95
Fat, g/100 g	9.3	9.0	2.6
Total dietary fiber, g/100 g	5.09	5.17	17.5
Dry matter, g/100 g	89.6	89.6	98.0

### Study Design

The study design was chosen to be in line with recommendations made by the World Health Organization (WHO) for performing an *in vivo* rat assay for true protein digestibility (6). After a 2-day acclimation period, the 10-day trial started. Rats were randomly assigned to either the nitrogen-free or test groups (*n* = 4 rats per group) based on body weight such that the group mean body weight did not vary by more than 5 g. Detailed observations were conducted for each animal one time during the acclimation phase and then one time daily during the trial. During the 10-day trial period, 15 g/d of each diet was offered so that feed intake could be measured. Full feeder weights were collected on days 5–9 of the trial, empty feeder weights were collected on days 6–10. Spilled feed was also collected and composited individually per animal for days 5–9. At the end of the study, spilled feed was air-dried for at least 3 days and weighed. The weights of uneaten and spilled feed were deducted from the weight of the feed offered to determine the total feed intake. Body weights were measured on days 1, 5, and 10 of the trial; feces were collected on days 6–10. Instances of coprophagy or urinary contamination were not measured.

### Fecal Analyses and True Protein Digestibility Calculation

Fresh feces were collected each day (days 6–10) and held at ambient temperature before being dried in a vacuum oven at 100°C. Feces were weighed, ground, and combined into a single sample per animal prior to the analysis of nitrogen content (AOCS Ac 4-91) and DM content (AOAC 925.09). For true protein digestibility, all variables were divided by DM intake to control for differing amounts of feed intake and DM between groups. The true protein digestibility score was calculated as:

$$\begin{array}{l} T=\frac{I-\left(F-Fk\right)\;}{I}x\;100 \end{array}$$

where *I* is equal to the nitrogen intake of the test group, *F* is equal to fecal nitrogen of the test group, and *Fk* (endogenous nitrogen) is equal to fecal nitrogen of the nitrogen-free group.

### Amino Acid Score and PDCAAS Calculations

Crude protein [AOAC (7) 968.06], tryptophan (AOAC 988.15), and other amino acids (8–11) were determined on raw walnuts (not defatted, Table 2). Amino acid scores (AAS) were estimated as:

$$\begin{array}{l} AAS=\frac{mg\;amino\;acid\;in\;1\;g\;of\;test\;protein\;}{mg\;of\;same\;amino\;acid\;in\;1\;g\;of\;reference\;pattern}x\;100 \end{array}$$

where the scoring patterns for the “child” (6 months to 3 years of age) and “older child, adolescent, and adult” (3–10 years of age) groups from Table 5 of the 2013 FAO Expert Consultation on Protein Quality Evaluation in Human Nutrition (12) were used as the reference pattern. PDCAAS was estimated by multiplying the lowest amino acid score by true protein digestibility. All scores were estimated using nitrogen conversion factors of 5.30 and 6.25.

**Table 2:** Analyzed amino acid composition of raw walnuts.

		**Crude protein estimate, g protein/100 g walnut**	
		***N* ×5.30**	***N* ×6.25**
		**13.2**	**15.6**
**Amino acid**	**mg AA/g walnut**	**mg AA/g protein**	**mg AA/g protein**
**Indispensable**			
Arginine	22.30	168.94	143.22
Histidine	2.87	21.74	18.43
Isoleucine	5.81	44.02	37.32
Leucine	10.50	79.55	67.44
Lysine	4.02	30.45	25.82
Methionine	2.38	18.03	15.29
Phenylalanine	6.40	48.48	41.10
Threonine	4.96	37.58	31.86
Tryptophan	1.41	10.68	9.06
Valine	6.47	49.02	41.55
**Dispensable**			
Alanine	6.24	47.27	40.08
Aspartic acid	14.00	106.06	89.92
Cystine	2.12	16.06	13.62
Glutamic acid	27.30	206.82	175.34
Glycine	7.13	54.02	45.79
Proline	5.39	40.83	34.62
Serine	7.57	14.01	48.62
Tyrosine	5.06	38.33	32.50
Sulfur amino acids (Met + Cys)	43.98	333.18	282.47
Aromatic AA (Phe, Tyr, Trp)	12.87	97.50	82.66

*N, nitrogen; AA, amino acid; Met, methionine; Cys, cystine; Phe, phenylalanine; Tyr, tyrosine; Trp, tryptophan*.

## Results

All animals completed the study as described and were transferred to a stock colony upon study completion. No adverse events or remarkable observations were reported. As expected, feed intake differed between groups with the test group consuming more feed (Table 3), resulting in the nitrogen-free group losing 14.5 ± 1.7 g (mean ± SD) and the test group gaining 5.5 ± 1.7 g throughout the trial (a mean difference of 20 g, *P* < 0.01). Using the mean and SD of metabolic nitrogen, it was observed that an effect size (Cohen's d) of 7.49 was achieved with a power (1-β) of 0.99 and an α of 0.01.

**Table 3:** True protein digestibility of defatted walnuts^a^.

**Group**	**Subject**	**Feed intake, g DM**	**Nitrogen intake, mg**	**mg Nitrogen intake/g DM intake**	**Calculated Fecal Nitrogen, mg**	**Metabolic Nitrogen, mg Fecal N/g DM intake**	**Corrected Metabolic Nitrogen, mg Fecal N/g DM intake**
Nitrogen-free	1	21.5	13.1	0.6	13.2	0.61	
Nitrogen-free	2	18.8	11.4	0.6	10.1	0.54	
Nitrogen-free	3	21.5	13.1	0.6	18.8	0.88	
Nitrogen-free	4	20.6	12.5	0.6	12.7	0.62	
Test	5	41.2	903.9	21.9	145.8	3.53	2.87
Test	6	43.0	943.2	21.9	192.7	4.48	3.82
Test	7	39.4	864.6	21.9	137.3	3.48	2.82
Test	8	43.0	943.2	21.9	138.8	3.23	2.57
Nitrogen-free (Mean ± SD)		20.6 ± 1.3	12.5 ± 0.8	0.6 ± 0.0	13.7 ± 3.7	**0.66** ± 0.15	
Test (Mean ± SD)		41.7 ± 1.7	913.7 ± 37.6	**21.9** ± 0.0	153.7 ± 26.3	**3.68** ± 0.55	3.02 ± 0.55
**True protein digestibility estimate**							
*I*		**21.9** ± 0.0					
*F*		**3.68** ± 0.55					
*Fk*		**0.66** ± 0.15					
TD		**86.22%** ± 1.83%					

a *True protein digestibility was estimated using the equation $TD\;=\;\frac{I-\left(F-Fk\right)}{I}$, where I is equal to nitrogen intake, F is equal to fecal nitrogen of the test diet, and Fk is equal to fecal nitrogen of the nitrogen-free diet. Estimates were corrected for dry matter intake. Bolded numbers indicate values used in the calculation of true protein digestibility*.

*N, nitrogen; DM, dry matter*.

True protein digestibility of the test diet was estimated to be 86.22 ± 1.83% (Table 3). Four AAS and PDCAAS were generated using protein conversion factors of *N* × 5.30 and *N* × 6.25, and two reference patterns (Table 4). The AAS ratios were determined to be: Child (*N* × 5.30), 0.53; Child (*N* × 6.25), 0.45; Adult (*N* × 5.30), 0.63; Adult (*N* × 6.25), 0.54. The PDCAAS were determined to be: Child (*N* × 5.30), 46%; Child (*N* × 6.25), 39%; Adult (*N* × 5.30), 55%; Adult (*N* × 6.25), 46%. Lysine was the limiting amino acid in all cases.

**Table 4:** Protein digestibility-corrected amino acid score (PDCAAS) of raw walnuts.

			**AAS ratios**			
	**Reference scoring pattern mg/g protein requirement**		**Child**		**Older child, adolescent, adult**	
**Amino acid**	**Child (6 months to 3 years)^a^**	**Older child, adolescent, adult^b^(3–10 years)**	N*5.30	N*6.25	N*5.30	N*6.25
Histidine	20	16	1.09	0.92	1.36	1.15
Isoleucine	32	30	1.38	1.17	1.47	1.24
Leucine	66	61	1.21	1.02	1.30	1.11
Lysine	57	48	**0.53**	**0.45**	**0.63**	**0.54**
Sulfur Amino Acids (Met + Cys)	27	23	12.34	10.46	14.49	12.28
Aromatic AA (Phe, Tyr, Trp)	52	41	1.88	1.59	2.38	2.02
Threonine	31	25	1.21	1.03	1.50	1.27
Tryptophan	8.5	7	1.26	1.07	1.62	1.37
Valine	43	40	1.14	0.97	1.23	1.04
**Population**	**Protein conversion**	**AAS**	**PDCAAS**			
Child	N*5.30	0.53	46%			
	N*6.25	0.45	39%			
Older child, adolescent, adult	N*5.30	0.63	55%			
	N*6.25	0.54	46%			

a, b *Scoring patterns for the child and older child, adolescent, and adult groups were taken from Table 5 of the 2013 FAO Expert Consultation on Protein Quality Evaluation in Human Nutrition. Bolded numbers indicate the AAS ratios used in the estimation of PDCAAS*.

*PDCAAS, protein digestibility corrected amino acid score; AAS, amino acid score; N, nitrogen; Met, methionine; Cys, cystine; Phe, phenylalanine; Tyr, tyrosine; Trp, tryptophan*.

## Discussion

In this study, we report the results of a dietary intervention trial in male Sprague Dawley rats consuming either a nitrogen-free diet or a diet composed of 12.28% protein (*N* × 6.25 on an as-is basis) from defatted walnuts to determine the PDCAAS for walnuts. The PDCAAS of walnuts for adults was 46 or 55%, and for children 39 or 46%, depending on the conversion factor. Regardless, this suggests that walnuts are not a quality source of protein, specifically limited by the concentration of lysine.

The FAO, WHO, and the US Food and Drug Administration (FDA) first published PDCAAS scoring in 1991 (6). PDCAAS is designed to allow for comparisons of whole-food protein source quality, where a score of 1 is indicative of a quality protein source, while a score of 0 is indicative of a poor-quality protein source (the absence of one or more essential amino acids). The quality of protein is typically higher in meat and dairy products (4, 13). However, some plant sources including soy and pea protein also have high PDCAAS (4). Among the nine types of tree nuts, a PDCAAS previously existed for only two of them: almonds and pistachios. While pistachios have high PDCAAS of 0.73 (raw) and 0.81 (roasted) (14), almonds have a lower score between 0.22 and 0.48 (depending on varietal), indicating that they are a low-quality source of protein (15, 16).

The provision of multiple PDCAAS in this report may be a point of confusion. The FDA Code of Federal Regulations 21 C.F.R. § 101.9 states that “Protein content may be calculated based on the factor 6.25 times the nitrogen content of the food as determined by the appropriate method of analysis as given in the ‘Official Methods of Analysis of the AOAC International,' except when official AOAC procedures described in this paragraph (c) (7) require a specific factor other than 6.25, that specific factor shall be used.” Bailey and Stein (14) provide the digestible indispensable amino acid score (DIAAS) of pistachios using conversion factors of both 5.30 and 6.25, noting that the AOAC recommends a conversion factor of 5.30 for “other nuts” (not peanuts, Brazil nuts, or almonds) to calculate crude protein on food labels. The FAO, on the other hand, suggests a conversion of 6.25 is used as a standard across all proteins (12). Furthermore, the FAO recommends that for regulatory purposes, reference amino acid scoring patterns for young children should be taken from children aged 6 months to 3 years, whereas for older children, adolescents, and adults, the pattern for 3- to 10-year-old children should be used. Hence, we estimated four PDCAAS using both conversion factors and two reference populations such that readers could use the appropriate score for their use case.

This study represents a step toward better understanding the protein quality of tree nuts. However, better metrics to evaluate protein quality do exist, such as the DIAAS, calculated from ileal digesta (12). Determining a DIAAS for walnuts may be pertinent, as some studies have shown that PDCAAS may overestimate the quality of some proteins, especially those of poor quality (17, 18). Currently, pistachios are the only tree nuts with an estimated DIAAS (14). The study in pistachios also highlights the differences in protein quality based on preparation. In this study, only defatted walnuts were evaluated. However, the method of preparation may impact the protein quality scores (PDCAAS or DIAAS); this could be a focus of future study. Additionally, the PDCAAS works presented in this study and on almonds (15, 16) were performed in rats, while the pistachio feeding trial was conducted in pigs, a model that better estimates human nutrition (19). There are notable limitations to using PDCAAS, including the sample collection site and the artificial truncation of scores. Specifically, estimated nitrogen in fecal samples includes that from microbial fermentation of protein, whereas the DIAAS uses true ileal digestibility using samples collected from the terminal ileum. Additionally, PDCAAS is limited to an artificial truncation of 1.0, underestimating the value of high-quality proteins where scores might be above 1.0. To date, many foods have been evaluated using PDCAAS, while limited (but increasing) numbers of foods have been assessed using DIAAS. Despite these limitations, the PDCAAS of walnuts has not been demonstrated previously, and future research should determine a DIAAS value for walnuts and other tree nuts to increase the comparability of these nutrient-dense foods.

## Conclusions

In this study, we have determined a PDCAAS for walnuts, as has been previously reported for pistachios and almonds. The true digestibility score for walnuts was determined to be 86.22 ± 1.83% and PDCAAS of 39 or 46% for children and 46 or 55% for adults, depending on the conversion factor used. Raw walnuts are not a good quality source of protein.

## Data Availability Statement

The raw data supporting the conclusions of this article will be made available by the authors, without undue reservation.

## Ethics Statement

The animal study was reviewed and approved by International Animal Care and Use Committee at Covance, Inc. (Princeton, NJ; protocol #8379737).

## Author Contributions

SF performed the analysis. KL wrote the first manuscript draft. Both authors contributed to manuscript revisions, have read, and approved the final manuscript.

## Conflict of Interest

The California Walnut Commission funded Covance Laboratories Inc. to execute the study and Traverse Science Inc. to interpret the data and write the manuscript. No members of either the California Walnut Commission or Covance Laboratories Inc. had a part in the interpretation of the data or preparation of the manuscript. SF and KL are employees of Traverse Science, Inc., of which SF has ownership.

## Publisher's Note

All claims expressed in this article are solely those of the authors and do not necessarily represent those of their affiliated organizations, or those of the publisher, the editors and the reviewers. Any product that may be evaluated in this article, or claim that may be made by its manufacturer, is not guaranteed or endorsed by the publisher.
